# Supplementary material for: The Evolution and Characterization of the RNA Interference Pathways in Lophotrochozoa
Source: Genome Biol Evol. 2024 May 7;16(5):evae098. doi: 10.1093/gbe/evae098 (PMC11114477; doi:10.1093/gbe/evae098)
Supplement: evae098_Supplementary_Data [file evae098_supplementary_data.zip › Supplementary_materials.zip!/selected_miRNAs/NoGe_selected_miRNAs/mirdeep_pdf_files/NMRB01000012.1_Notospermus_geniculatus_strain_Ushimado_scaffold12,_whole_genome_shotgun_sequence_565.pdf]

Provisional ID	:	NMRB01000012.1_Notospermus_geniculatus_strain_Ushimado_scaffold12,_whole_genome_shotgun_sequence_565
Score total	:	37685.4
Score for star read(s)	:	3.9
Score for read counts	:	37676.7
Score for mfe	:	1.7
Score for randfold	:	0
Score for cons. seed	:	3
Total read count	:	73913
Mature read count	:	73389
Loop read count	:	0
Star read count	:	524

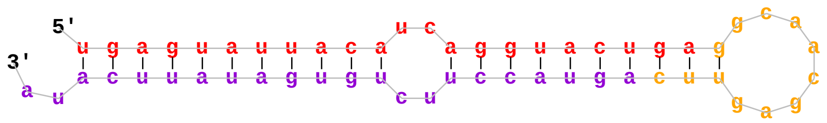

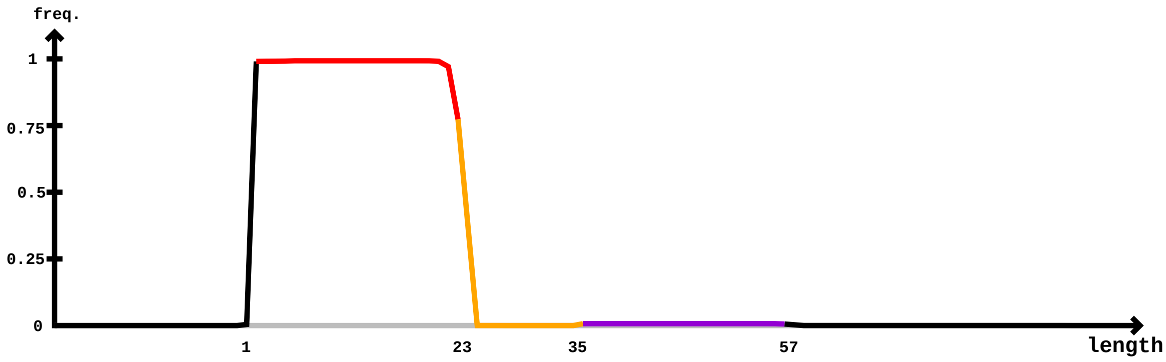

Mature **Star**

```
5'- aaaguagacuucucaguuuugaguauuacaucagguacugaggcaacgaguucaguaccuucugugauauucauaacuuggaaguuguagacagcaggaagaccaacaucu  -3'   obs
    aaaguagacuucucaguuuugaguauuacaucagguacugaggcaacgaguucaguaccuucugugauauucauaacuuggaaguuguagacagcaggaagaccaacaucu         exp
    ......((((((.((((.((((((((((..(((((((((........)))))))))..)))))))))).)))).)))))))..........((....)).............  reads   mm   sample
    .aaguagacuucuUaguuu............................................................................................  1       1    Fm3
    .................uugaguauuacaucagguacu.........................................................................  3       0    Fm3
    .................uugaguauuacaucagguacug........................................................................  39      0    Fm3
    .................uugaguauuacaucagguacugU.......................................................................  128     1    Fm3
    ..................ugaguauuacaucagguCc..........................................................................  2       1    Fm3
    ..................Cgaguauuacaucagguac..........................................................................  1       1    Fm3
    ..................ugaguauuacaucaggAac..........................................................................  1       1    Fm3
    ..................ugaguauuacaucagguac..........................................................................  91      0    Fm3
    ..................ugaguNuuacaucagguac..........................................................................  2       1    Fm3
    ..................ugaguauuacauUagguacu.........................................................................  1       1    Fm3
    ..................ugaguauuacaucagguUcu.........................................................................  1       1    Fm3
    ..................ugaguauuacaucagguacu.........................................................................  469     0    Fm3
    ..................ugaguauuacaucagguCcu.........................................................................  3       1    Fm3
    ..................ugaguauuacUucagguacu.........................................................................  1       1    Fm3
    ..................ugaguauuacGucagguacu.........................................................................  2       1    Fm3
    ..................ugaguNuuacaucagguacu.........................................................................  1       1    Fm3
    ..................ugaguauuacaucaggGacu.........................................................................  2       1    Fm3
    ..................ugGguauuacaucagguacu.........................................................................  1       1    Fm3
    ..................ugaguauuacauUagguacug........................................................................  2       1    Fm3
    ..................ugaguaGuacaucagguacug........................................................................  1       1    Fm3
    ..................ugaguauuacaucagguUcug........................................................................  9       1    Fm3
    ..................ugaguauuacaucagguacCg........................................................................  7       1    Fm3
    ..................ugaguCuuacaucagguacug........................................................................  1       1    Fm3
    ..................ugaguauuacaucGgguacug........................................................................  1       1    Fm3
    ..................ugaguauuacaucagguacuC........................................................................  1       1    Fm3
    ..................ugaguauuacaAcagguacug........................................................................  1       1    Fm3
    ..................ugaguauuacaucaggAacug........................................................................  14      1    Fm3
    ..................ugaguauuacauAagguacug........................................................................  1       1    Fm3
    ..................ugaAuauuacaucagguacug........................................................................  1       1    Fm3
    ..................ugaguauuacUucagguacug........................................................................  3       1    Fm3
    ..................ugaguauuacaucagguacug........................................................................  5863    0    Fm3
    ..................ugaguauuacaucagguGcug........................................................................  25      1    Fm3
    ..................ugaguauCacaucagguacug........................................................................  6       1    Fm3
```

```
                   Mature                            Star
aaaguagacuucucaguuuugaguauuacaucagguacugaggcaacgaguucaguaccuucugugauauucauaacuuggaaguuguagacagcaggaagaccaacaucu
...................ugaguauuacaucagguaUug.......................................................................   8       1   Fm3
...................ugaguNuuacaucagguacug.......................................................................   1       1   Fm3
...................ugaguauuacaucaggGacug.......................................................................   12      1   Fm3
...................ugaguauuacaucagCuacug.......................................................................   3       1   Fm3
...................ugaguauuacaucaggCacug.......................................................................   14      1   Fm3
...................ugagCauuacaucagguacug.......................................................................   1       1   Fm3
...................ugaguauuacaucagguacuU.......................................................................   2       1   Fm3
...................ugaguauuacaucagguCcug.......................................................................   26      1   Fm3
...................ugaguauuacCucagguacug.......................................................................   3       1   Fm3
...................Ngaguauuacaucagguacug.......................................................................   3       1   Fm3
...................ugCguauuacaucagguacug.......................................................................   2       1   Fm3
...................ugaguauuacaucagguacuA.......................................................................   2       1   Fm3
...................ugaguaCuacaucagguacug.......................................................................   1       1   Fm3
...................uAaguauuacaucagguacug.......................................................................   5       1   Fm3
...................ugaguauuacaucaUguacug.......................................................................   4       1   Fm3
...................ugaguauuacGucagguacug.......................................................................   9       1   Fm3
...................ugaguauuaAaucagguacug.......................................................................   1       1   Fm3
...................ugGguauuacaucagguacug.......................................................................   4       1   Fm3
...................ugaguauuaUaucagguacug.......................................................................   1       1   Fm3
...................uUaguauuacaucagguacug.......................................................................   1       1   Fm3
...................Cgaguauuacaucagguacug.......................................................................   2       1   Fm3
...................ugaguauuacaucagAuacug.......................................................................   1       1   Fm3
...................ugaguauuacaucGgguacuga......................................................................   1       1   Fm3
...................ugaguauuacaucagCuacuga......................................................................   2       1   Fm3
...................ugaguauuacCucagguacuga......................................................................   5       1   Fm3
...................uAaguauuacaucagguacuga......................................................................   7       1   Fm3
...................ugaguauuacauGagguacuga......................................................................   1       1   Fm3
...................ugaguauuacaucagguaUuga......................................................................   1       1   Fm3
...................ugaguauuacaucagguacAga......................................................................   1       1   Fm3
...................ugaguauuGcaucagguacuga......................................................................   1       1   Fm3
...................ugaguauuacaucaggCacuga......................................................................   26      1   Fm3
...................Cgaguauuacaucagguacuga......................................................................   1       1   Fm3
...................ugaguauuacaucCgguacuga......................................................................   1       1   Fm3
...................ugaguGuuacaucagguacuga......................................................................   2       1   Fm3
...................ugaguauuacaucaUguacuga......................................................................   1       1   Fm3
...................ugagAauuacaucagguacuga......................................................................   1       1   Fm3
...................ugaguauuacaucaggGacuga......................................................................   22      1   Fm3
...................ugaguauuacUucagguacuga......................................................................   2       1   Fm3
...................ugaguaCuacaucagguacuga......................................................................   3       1   Fm3
...................ugaguUuuacaucagguacuga......................................................................   3       1   Fm3
...................ugaguauuacaAcagguacuga......................................................................   1       1   Fm3
...................ugaguauuacauUagguacuga......................................................................   8       1   Fm3
...................ugaguNuuacaucagguacuga......................................................................   1       1   Fm3
...................ugaguauuaUaucagguacuga......................................................................   11      1   Fm3
...................ugaguauuacaucagguacugG......................................................................   1       1   Fm3
...................ugaguauuacGucagguacuga......................................................................   15      1   Fm3
...................ugaguauuCcaucagguacuga......................................................................   1       1   Fm3
...................ugagCauuacaucagguacuga......................................................................   5       1   Fm3
...................ugaguauuacaucagAuacuga......................................................................   2       1   Fm3
...................ugaguauuacaucagguacugU......................................................................   21371   1   Fm3
...................ugaUuauuacaucagguacuga......................................................................   5       1   Fm3
...................ugCguauuacaucagguacuga......................................................................   2       1   Fm3
...................ugaguauuacaCcagguacuga......................................................................   3       1   Fm3
...................ugaguauuacaucaggAacuga......................................................................   10      1   Fm3
...................ugaguauuacaucagguacuga......................................................................   8093    0   Fm3
...................ugaguauuacaucagguacuAa......................................................................   3       1   Fm3
...................uCaguauuacaucagguacuga......................................................................   1       1   Fm3
...................uUaguauuacaucagguacuga......................................................................   5       1   Fm3
...................ugaguauuacaucagguCcuga......................................................................   30      1   Fm3
...................ugaguauuacaucagguUcuga......................................................................   17      1   Fm3
...................ugaguaGuacaucagguacuga......................................................................   1       1   Fm3
...................ugaguauuacauAagguacuga......................................................................   1       1   Fm3
...................ugaguauuacaucagguacugC......................................................................   10      1   Fm3
...................ugaguauuUcaucagguacuga......................................................................   3       1   Fm3
...................ugUguauuacaucagguacuga......................................................................   1       1   Fm3
...................ugaguauuacaucagguGcuga......................................................................   32      1   Fm3
...................ugGguauuacaucagguacuga......................................................................   5       1   Fm3
...................Ngaguauuacaucagguacuga......................................................................   5       1   Fm3
...................ugaguauuacaucagguacugaC.....................................................................   3       1   Fm3
...................ugaguauuacaucagguacugaA.....................................................................   93      1   Fm3
```

```
                   Mature                          Star
aaaguagacuucucaguuuugaguauuacaucagguacugaggcaacgaguucaguaccuucugugauauucauaacuuggaaguuguagacagcaggaagaccaacaucu
...................ugaguauuacaucagguacugaU.....................................................................    1482    1    Fm3
...................ugaguauuacaucagguacugag.....................................................................    1       0    Fm3
...................ugaguauuacaucagguacCgaggc...................................................................    1       1    Fm3
...................ugaguauuacaucagguacugaggca..................................................................    2       0    Fm3
....................gaguauuacaucagguacug.......................................................................    6       0    Fm3
....................Uaguauuacaucagguacug.......................................................................    1       1    Fm3
....................gaguauuacaucagguacugU......................................................................    5       1    Fm3
....................gaguauuacaucagguacuga......................................................................    6       0    Fm3
....................gaguauuacaGcagguacuga......................................................................    1       1    Fm3
....................gaguauuacaucagguacugaU.....................................................................    1       1    Fm3
.....................aguauuacaucagguacAga......................................................................    1       1    Fm3
.....................aguauuacauAagguacuga......................................................................    1       1    Fm3
.....................Uguauuacaucagguacuga......................................................................    1       1    Fm3
.....................aguauuacaucagguacuga......................................................................    10      0    Fm3
......................guauuacaucagguacuga......................................................................    5       0    Fm3
......................guauuacaucagguacugaU.....................................................................    1       1    Fm3
.......................uauuacaucagguacugU......................................................................    2       1    Fm3
.......................uauuacaucagguacuCa......................................................................    1       1    Fm3
.......................uauuacaucagguacuga......................................................................    69      0    Fm3
.......................uauuacaucagguacugaU.....................................................................    2       1    Fm3
.....................................................aguaccuucugugauauuc.......................................    4       0    Fm3
.....................................................aguaccuucugugauauucau.....................................    15      0    Fm3
.....................................................aguaccuucugugauauCcau.....................................    1       1    Fm3
.....................................................aguaccuucugugauauucauU....................................    2       1    Fm3
.....................................................aguaccuucugugauauucaua....................................    53      0    Fm3
.....................................................aguaccuucugugauauAcaua....................................    1       1    Fm3
.....................................................aguaccuucugugauauucauaa...................................    1       0    Fm3
......................................................guaccuucugugauauucaua....................................    3       0    Fm3
.......................................................uaccuucugugauauucauaaA..................................    2       1    Fm3

..................uugaguauuacaucagguacug.......................................................................    1       0    Mm3
..................uugaguauuacaucagguacugG......................................................................    1       1    Mm3
..................Cugaguauuacaucagguacuga......................................................................    1       1    Mm3
..................uugaguauuacaucagguacugU......................................................................    12      1    Mm3
...................ugaguauuacaucagguac.........................................................................    9       0    Mm3
...................ugaguauuacaucagguacG........................................................................    2       1    Mm3
...................ugaguauuacaucagguacu........................................................................    149     0    Mm3
...................ugGguauuacaucagguacu........................................................................    1       1    Mm3
...................ugaguauuacaucaggGacug.......................................................................    2       1    Mm3
...................ugaguauuacaucagguGcug.......................................................................    2       1    Mm3
...................ugaguauuacaucagguacug.......................................................................    301     0    Mm3
...................ugaguauuacCucagguacug.......................................................................    1       1    Mm3
...................ugaguauuacaucaggCacug.......................................................................    2       1    Mm3
...................ugaguauuacauUagguacug.......................................................................    1       1    Mm3
...................Cgaguauuacaucagguacuga......................................................................    2       1    Mm3
...................ugaguauuacaucaggGacuga......................................................................    2       1    Mm3
...................ugaguauuacaucaggCacuga......................................................................    2       1    Mm3
...................ugaguauuacaucagguUcuga......................................................................    1       1    Mm3
...................uAaguauuacaucagguacuga......................................................................    2       1    Mm3
...................ugaguauuacaucagguacuga......................................................................    756     0    Mm3
...................ugaguauuacaucaggAacuga......................................................................    1       1    Mm3
...................ugaguauuacGucagguacuga......................................................................    2       1    Mm3
...................ugaguauuacaucagguacugC......................................................................    5       1    Mm3
...................ugaguauuacaucagguacugU......................................................................    2114    1    Mm3
...................ugaguauuacaucagguacugG......................................................................    16      1    Mm3
...................ugaguauuacaucagguacCga......................................................................    1       1    Mm3
...................ugaguauuacaucagguCcuga......................................................................    2       1    Mm3
...................ugaguauuacaucagguGcuga......................................................................    4       1    Mm3
...................ugaguauuacaucagguaAuga......................................................................    1       1    Mm3
...................ugaguauuaUaucagguacuga......................................................................    2       1    Mm3
...................ugaguauuacaucagguacugaA.....................................................................    24      1    Mm3
...................ugaguauuacaucagguacugag.....................................................................    1       0    Mm3
...................ugaguauuacaucagguacugaU.....................................................................    343     1    Mm3
....................gaguauuacaucagguacuC.......................................................................    1       1    Mm3
......................guauuacaucagguacuga......................................................................    1       0    Mm3
......................guauuacaucagguacugaU.....................................................................    1       1    Mm3
.......................uauuacaucagguacuga......................................................................    2       0    Mm3
.....................................................aguaccuucugugauauucau.....................................    4       0    Mm3
.....................................................aguaccuucugugauauucauU....................................    2       1    Mm3
.....................................................aguaccuucugugauauCcaua....................................    1       1    Mm3
```

```
                   Mature                 Star
aaaguagacuucucaguuuugaguauuacaucagguacugaggcaacgaguucaguaccuucugugauauucauaacuuggaaguuguagacagcaggaagaccaacaucu
.....................................................aguaccuucugugauauucaua....................................   57     0   Mm3
.....................................................aguaccuucuCugauauucaua....................................   1      1   Mm3

..................uugaguauuacaucagguacu........................................................................   5      0   Fm2
..................uugaguauuacaucaggAacug.......................................................................   1      1   Fm2
..................uugaguauuacaucagguacug.......................................................................   20     0   Fm2
..................uugaguauuacaucaggGacug.......................................................................   1      1   Fm2
..................Cugaguauuacaucagguacuga......................................................................   2      1   Fm2
...................ugaguauuacaucagguac.........................................................................   5      0   Fm2
...................ugaguauuacaucagguCcu........................................................................   1      1   Fm2
...................ugaguauuacaucagguGcu........................................................................   1      1   Fm2
...................ugaguauuacaucagguacG........................................................................   1      1   Fm2
...................ugaguauuacaucagguacu........................................................................   148    0   Fm2
...................ugaguauuacaucagguGcug.......................................................................   18     1   Fm2
...................ugaguauuacaucagguacug.......................................................................   2777   0   Fm2
...................ugaCuauuacaucagguacug.......................................................................   1      1   Fm2
...................ugaguauuacaucagCuacug.......................................................................   1      1   Fm2
...................ugaguauuacaucagguUcug.......................................................................   8      1   Fm2
...................ugaguauuUcaucagguacug.......................................................................   1      1   Fm2
...................ugGguauuacaucagguacug.......................................................................   2      1   Fm2
...................ugaguauuacauUagguacug.......................................................................   1      1   Fm2
...................ugaguauuacaucagguacGg.......................................................................   10     1   Fm2
...................ugaguauuacaucagguacuA.......................................................................   6      1   Fm2
...................ugCguauuacaucagguacug.......................................................................   1      1   Fm2
...................ugaguauuacaucaggCacug.......................................................................   5      1   Fm2
...................ugaguauuacaucagguCcug.......................................................................   9      1   Fm2
...................ugagCauuacaucagguacug.......................................................................   3      1   Fm2
...................ugaguauuacUucagguacug.......................................................................   1      1   Fm2
...................ugaguauuacaucagguacuU.......................................................................   1      1   Fm2
...................ugaguauuaAaucagguacug.......................................................................   1      1   Fm2
...................ugaguauuacaucaggGacug.......................................................................   8      1   Fm2
...................ugaguauuacaucaggAacug.......................................................................   7      1   Fm2
...................ugaguauuacGucagguacug.......................................................................   2      1   Fm2
...................Cgaguauuacaucagguacug.......................................................................   2      1   Fm2
...................Ngaguauuacaucagguacug.......................................................................   4      1   Fm2
...................ugaguauuacCucagguacug.......................................................................   4      1   Fm2
...................ugaguauuacaucaUguacug.......................................................................   2      1   Fm2
...................ugaguauuacaucaggAacuga......................................................................   6      1   Fm2
...................ugaguauuacaucaggCacuga......................................................................   2      1   Fm2
...................ugaguauuacaucagguacugG......................................................................   94     1   Fm2
...................ugaguauuacaucagguacuUa......................................................................   1      1   Fm2
...................ugaguauAacaucagguacuga......................................................................   1      1   Fm2
...................ugaguauuacaucaggGacuga......................................................................   5      1   Fm2
...................ugUguauuacaucagguacuga......................................................................   1      1   Fm2
...................ugaguauuacGucagguacuga......................................................................   1      1   Fm2
...................ugaguauuacaucagguacugU......................................................................   2902   1   Fm2
...................ugaguauuacaucagguacuga......................................................................   1372   0   Fm2
...................ugaUuauuacaucagguacuga......................................................................   1      1   Fm2
...................ugaguGuuacaucagguacuga......................................................................   1      1   Fm2
...................ugaguauuacaucaCguacuga......................................................................   1      1   Fm2
...................ugaguauuUcaucagguacuga......................................................................   1      1   Fm2
...................ugaguauuacaucagguaGuga......................................................................   1      1   Fm2
...................ugaguauuacaucagguCcuga......................................................................   8      1   Fm2
...................ugagCauuacaucagguacuga......................................................................   1      1   Fm2
...................Ngaguauuacaucagguacuga......................................................................   2      1   Fm2
...................ugaguauuacCucagguacuga......................................................................   1      1   Fm2
...................ugaguauuacaucagguGcuga......................................................................   4      1   Fm2
...................ugaguauuacaucagguUcuga......................................................................   1      1   Fm2
...................ugaguauuacaucagguacugaA.....................................................................   11     1   Fm2
...................ugaguauuacaucagguacugUg.....................................................................   3      1   Fm2
...................ugaguauuacaucagguacugaU.....................................................................   217    1   Fm2
....................gaguauuacaucagguacug.......................................................................   1      0   Fm2
....................gaguauuacaucagguacugU......................................................................   3      1   Fm2
......................guauuacaucagguacugU......................................................................   2      1   Fm2
.....................................................aguaccuucugugauauuca......................................   4      0   Fm2
.....................................................aguaccuucugugauaGucau.....................................   1      1   Fm2
.....................................................aguaccuucugugauauucau.....................................   20     0   Fm2
.....................................................Uguaccuucugugauauucaua....................................   2      1   Fm2
.....................................................aguaccuuAugugauauucaua....................................   1      1   Fm2
.....................................................aguaccuucugugauauucaua....................................   147    0   Fm2
```

```
                   Mature                          Star
aaaguagacuucucaguuuugaguauuacaucagguacugaggcaacgaguucaguaccuucugugauauucauaacuuggaaguuguagacagcaggaagaccaacaucu
.....................................................aguaccuucugugauauucauU....................................    9     1    Fm2
.....................................................aguaccuucugugauauAcaua....................................    1     1    Fm2

..................uugaguauuacaucagguacu........................................................................    8     0    Fm1
..................uugaguauuacaucaggAacug.......................................................................    1     1    Fm1
..................uugaguauuacaucagguacug.......................................................................    23    0    Fm1
..................uugaguauuacaucagguacugU......................................................................    47    1    Fm1
...................ugaguauuacaucaggua..........................................................................    2     0    Fm1
...................ugaguauuacaucagguU..........................................................................    2     1    Fm1
...................ugaguauuacaucagguGc.........................................................................    2     1    Fm1
...................ugaguauuacaucagguac.........................................................................    26    0    Fm1
...................ugaguauuacaucagguacu........................................................................    435   0    Fm1
...................Ngaguauuacaucagguacu........................................................................    1     1    Fm1
...................ugaguauuacCucagguacu........................................................................    1     1    Fm1
...................ugGguauuacaucagguacu........................................................................    1     1    Fm1
...................ugaguauuacaucagguUcu........................................................................    2     1    Fm1
...................ugaguauuacaucagguacA........................................................................    1     1    Fm1
...................uCaguauuacaucagguacu........................................................................    1     1    Fm1
...................ugaguauuacaucagguGcu........................................................................    2     1    Fm1
...................ugaguNuuacaucagguacu........................................................................    1     1    Fm1
...................ugaguauuacaucagguacG........................................................................    7     1    Fm1
...................Cgaguauuacaucagguacug.......................................................................    1     1    Fm1
...................ugaguauuGcaucagguacug.......................................................................    1     1    Fm1
...................ugaguauuacaucaggCacug.......................................................................    11    1    Fm1
...................ugaguauuUcaucagguacug.......................................................................    2     1    Fm1
...................ugagGauuacaucagguacug.......................................................................    1     1    Fm1
...................ugaguauuacaucaggAacug.......................................................................    6     1    Fm1
...................ugaguauuacaucagguacCg.......................................................................    1     1    Fm1
...................ugagCauuacaucagguacug.......................................................................    3     1    Fm1
...................ugCguauuacaucagguacug.......................................................................    5     1    Fm1
...................ugaguauCacaucagguacug.......................................................................    1     1    Fm1
...................ugaguauuacaucagguUcug.......................................................................    5     1    Fm1
...................Ngaguauuacaucagguacug.......................................................................    3     1    Fm1
...................ugNguauuacaucagguacug.......................................................................    1     1    Fm1
...................ugaguauuacaucagguacuA.......................................................................    2     1    Fm1
...................ugaguauuCcaucagguacug.......................................................................    4     1    Fm1
...................ugaguauuacaucGgguacug.......................................................................    1     1    Fm1
...................ugaguauuacaucagguacGg.......................................................................    14    1    Fm1
...................ugaguUuuacaucagguacug.......................................................................    1     1    Fm1
...................ugaguauuacaucagguGcug.......................................................................    12    1    Fm1
...................ugaguauuacUucagguacug.......................................................................    4     1    Fm1
...................ugaguauuacCucagguacug.......................................................................    1     1    Fm1
...................ugaguauuacaucagguCcug.......................................................................    13    1    Fm1
...................ugaguauuacaucaggGacug.......................................................................    6     1    Fm1
...................ugaguauuacGucagguacug.......................................................................    4     1    Fm1
...................ugaguauuacaucagguacuC.......................................................................    1     1    Fm1
...................ugaguauuacaucagguacug.......................................................................    2983  0    Fm1
...................uUaguauuacaucagguacug.......................................................................    1     1    Fm1
...................ugaguauuacaucagguacuU.......................................................................    1     1    Fm1
...................ugaguGuuacaucagguacug.......................................................................    1     1    Fm1
...................uAaguauuacaucagguacug.......................................................................    1     1    Fm1
...................ugaguNuuacaucagguacug.......................................................................    1     1    Fm1
...................ugaguauuaAaucagguacuga......................................................................    1     1    Fm1
...................ugaguauuacaucagguCcuga......................................................................    9     1    Fm1
...................ugaguauuacGucagguacuga......................................................................    4     1    Fm1
...................ugaguauuacaucUgguacuga......................................................................    1     1    Fm1
...................ugaguauuacaucaggGacuga......................................................................    3     1    Fm1
...................ugaguauuacCucagguacuga......................................................................    2     1    Fm1
...................ugaguauuacaucagguacuga......................................................................    1818  0    Fm1
...................ugaguauuacaucagguacugG......................................................................    167   1    Fm1
...................ugaguauuacaucagguGcuga......................................................................    3     1    Fm1
...................ugUguauuacaucagguacuga......................................................................    1     1    Fm1
...................ugaguauuacaucagguacugU......................................................................    6012  1    Fm1
...................ugaguauuacauUagguacuga......................................................................    2     1    Fm1
...................ugaguauuacaucaggAacuga......................................................................    3     1    Fm1
...................ugaguauuacaucagguUcuga......................................................................    6     1    Fm1
...................ugaguauuacaucGgguacuga......................................................................    1     1    Fm1
...................ugaguauuacUucagguacuga......................................................................    1     1    Fm1
...................ugaguauuacaucagguacugC......................................................................    7     1    Fm1
...................ugaguauuaUaucagguacuga......................................................................    9     1    Fm1
```

```
                   Mature                 Star
aaaguagacuucucaguuuugaguauuacaucagguacugaggcaacgaguucaguaccuucugugauauucauaacuuggaaguuguagacagcaggaagaccaacaucu
...................ugaguNuuacaucagguacuga......................................................................   1     1    Fm1
...................ugaguauuacaucagguacCga......................................................................   1     1    Fm1
...................ugaguauuacaucaggCacuga......................................................................   6     1    Fm1
...................ugaguauuacaucagguacugaC.....................................................................   2     1    Fm1
...................ugaguauuacaucagguacugaA.....................................................................   19    1    Fm1
...................ugaguauuacaucagguacugaU.....................................................................   433   1    Fm1
...................ugaguauuacaucagguacugag.....................................................................   3     0    Fm1
...................ugaguauuacaucagguacugaUg....................................................................   1     1    Fm1
....................gaguauuacaucagguacu........................................................................   1     0    Fm1
....................gaguauuacaGcagguacug.......................................................................   1     1    Fm1
....................gaguauuacaucagguacugU......................................................................   1     1    Fm1
......................guauuacaucagguacuga......................................................................   5     0    Fm1
.......................uauuacaucagguacuga......................................................................   4     0    Fm1
.....................................................aguaccuucugugauauuc.......................................   3     0    Fm1
.....................................................aguaccuucugugauauuca......................................   3     0    Fm1
.....................................................aguaccuucuAugauauucau.....................................   1     1    Fm1
.....................................................aguaccuucugugauauucau.....................................   18    0    Fm1
.....................................................aguaccuucugugauauucauU....................................   2     1    Fm1
.....................................................aguaccuucugugauauucaua....................................   37    0    Fm1
...........................................................................acuuggaaguuguagacagcaggaagacc.......   4     0    Fm1

..................uugaguauuacaucagguacug.......................................................................   5     0    Mm2
..................uugaguauuacaucagguacugU......................................................................   10    1    Mm2
...................ugaguauuacaucagguac.........................................................................   9     0    Mm2
...................ugaguaNuacaucagguacu........................................................................   1     1    Mm2
...................ugaguauuacaucagguGcu........................................................................   2     1    Mm2
...................ugaguauuacaucaggGacu........................................................................   2     1    Mm2
...................ugaguauuacaucagguacu........................................................................   147   0    Mm2
...................ugaguauuacaucagguacG........................................................................   1     1    Mm2
...................ugaguauuacaucaggAacu........................................................................   1     1    Mm2
...................ugaguauuacaucagguacuA.......................................................................   1     1    Mm2
...................ugaguauuacaucaggCacug.......................................................................   5     1    Mm2
...................ugGguauuacaucagguacug.......................................................................   1     1    Mm2
...................ugaguauuacaucagguacGg.......................................................................   3     1    Mm2
...................uUaguauuacaucagguacug.......................................................................   1     1    Mm2
...................ugaguaNuacaucagguacug.......................................................................   1     1    Mm2
...................ugaguauuacaucaggGacug.......................................................................   2     1    Mm2
...................ugaguauuacaucaggAacug.......................................................................   1     1    Mm2
...................ugaguauuacaucagguacug.......................................................................   634   0    Mm2
...................ugaguauuacCucagguacug.......................................................................   1     1    Mm2
...................ugaguauuacaucagguGcug.......................................................................   3     1    Mm2
...................ugaguauuCcaucagguacug.......................................................................   1     1    Mm2
...................ugaguauuacaucagguCcug.......................................................................   2     1    Mm2
...................ugagCauuacaucagguacug.......................................................................   1     1    Mm2
...................ugCguauuacaucagguacug.......................................................................   1     1    Mm2
...................ugaguauuacaucagguacugU......................................................................   1814  1    Mm2
...................ugaguauuaUaucagguacuga......................................................................   1     1    Mm2
...................ugaguauuacaucagguCcuga......................................................................   2     1    Mm2
...................ugaguauuacaucagguacugG......................................................................   31    1    Mm2
...................ugaguauuacaucagguacuAa......................................................................   1     1    Mm2
...................ugagGauuacaucagguacuga......................................................................   1     1    Mm2
...................Ngaguauuacaucagguacuga......................................................................   2     1    Mm2
...................ugaguauuacaucaggAacuga......................................................................   1     1    Mm2
...................ugaguauuacaucagguGcuga......................................................................   5     1    Mm2
...................ugaguauuacaucaggGacuga......................................................................   4     1    Mm2
...................ugaguauuacaucagguaUuga......................................................................   3     1    Mm2
...................ugaguauGacaucagguacuga......................................................................   2     1    Mm2
...................ugaUuauuacaucagguacuga......................................................................   2     1    Mm2
...................ugaguauuacaucagAuacuga......................................................................   1     1    Mm2
...................ugaguauuacCucagguacuga......................................................................   1     1    Mm2
...................ugaguauuacauUagguacuga......................................................................   1     1    Mm2
...................ugaguauuacUucagguacuga......................................................................   1     1    Mm2
...................ugaguauuacaucagguUcuga......................................................................   1     1    Mm2
...................ugaguauuacaucagguacugC......................................................................   7     1    Mm2
...................ugaguauuacaucagguacuga......................................................................   831   0    Mm2
...................ugaguauuacaucagguacugaA.....................................................................   20    1    Mm2
...................ugaguauuacaucagguacugag.....................................................................   1     0    Mm2
...................ugaguauuacaucagguacugaU.....................................................................   144   1    Mm2
....................gaguauuacaucagguacug.......................................................................   1     0    Mm2
....................gaguauuacaucagguacuga......................................................................   1     0    Mm2
```

```
                    Mature                          Star
aaaguagacuucucaguuuugaguauuacaucagguacugaggcaacgaguucaguaccuucugugauauucauaacuuggaaguuguagacagcaggaagaccaacaucu
.....................aguauuacaucagguacuga......................................................................   1      0    Mm2
......................guauuacaucagguacuga......................................................................   2      0    Mm2
.......................uauuacaucagguacuga......................................................................   8      0    Mm2
.........................................ggcaacgaguucaguaccuucugugauauucaua....................................   2      0    Mm2
....................................................caguaccuucugugauauucau.....................................   1      0    Mm2
.....................................................aguaccuucugugauauuc.......................................   1      0    Mm2
.....................................................aguaccuucugugauauuca......................................   3      0    Mm2
.....................................................aguaccuucugugauGuucau.....................................   1      1    Mm2
.....................................................aguaccuucugugauauucau.....................................   11     0    Mm2
.....................................................aguaccuucugugauaAucau.....................................   1      1    Mm2
.....................................................aguaccuucugugauauCcaua....................................   1      1    Mm2
.....................................................aguaccuucugugauaGucaua....................................   1      1    Mm2
.....................................................aguaccuucugugauauucaua....................................   68     0    Mm2
.....................................................aguaccuucugugauauGcaua....................................   1      1    Mm2
.....................................................aguaccuucugugauauucauU....................................   1      1    Mm2
.....................................................aguaccuucugugauauAcaua....................................   1      1    Mm2
......................................................guaccuucugugauauucaua....................................   2      0    Mm2
...........................................................................acuuggaaguuguagacagcaggaagac........   1      0    Mm2

aaaguagacuucucaguuu............................................................................................   1      0    Mm1
...................ugaguauuacaucagguac.........................................................................   3      0    Mm1
...................ugaguauuacaucagguacu........................................................................   66     0    Mm1
...................ugaguauuacaCcagguacu........................................................................   1      1    Mm1
...................ugaguauuacaucagguCcu........................................................................   1      1    Mm1
...................ugaguauuacGucagguacug.......................................................................   3      1    Mm1
...................ugaguauuacaucagguacug.......................................................................   1457   0    Mm1
...................ugaguauuacaucagguGcug.......................................................................   6      1    Mm1
...................ugaguauCacaucagguacug.......................................................................   1      1    Mm1
...................ugaguauuacaucaggGacug.......................................................................   8      1    Mm1
...................ugagCauuacaucagguacug.......................................................................   1      1    Mm1
...................ugaguauuUcaucagguacug.......................................................................   1      1    Mm1
...................ugaguauuacCucagguacug.......................................................................   1      1    Mm1
...................ugaguauuacaucaggAacug.......................................................................   3      1    Mm1
...................uAaguauuacaucagguacug.......................................................................   2      1    Mm1
...................ugaguauuacaucagguCcug.......................................................................   3      1    Mm1
...................ugagGauuacaucagguacug.......................................................................   1      1    Mm1
...................ugaguauuacaucagguacGg.......................................................................   5      1    Mm1
...................ugaguauuacaucagguUcug.......................................................................   3      1    Mm1
...................ugaguauuacaucaggCacug.......................................................................   5      1    Mm1
...................ugaguauuacaucagguGcuga......................................................................   4      1    Mm1
...................ugaguauuacaucagguCcuga......................................................................   1      1    Mm1
...................ugUguauuacaucagguacuga......................................................................   1      1    Mm1
...................ugaguauuacaucagguacugG......................................................................   10     1    Mm1
...................ugaguauuacGucagguacuga......................................................................   1      1    Mm1
...................uCaguauuacaucagguacuga......................................................................   1      1    Mm1
...................ugaguauuacaucagguacugU......................................................................   4711   1    Mm1
...................ugCguauuacaucagguacuga......................................................................   1      1    Mm1
...................ugaguauuacaucagguUcuga......................................................................   3      1    Mm1
...................ugaguauuacaucGgguacuga......................................................................   1      1    Mm1
...................ugaguauuacaucagguacuga......................................................................   1227   0    Mm1
...................ugaguauuacCucagguacuga......................................................................   1      1    Mm1
...................ugaguauuacUucagguacuga......................................................................   1      1    Mm1
...................ugaguauuacaucUgguacuga......................................................................   1      1    Mm1
...................ugaguauuacaucagguacuUa......................................................................   1      1    Mm1
...................ugaguauuacaucaggAacuga......................................................................   3      1    Mm1
...................ugaguauuacauGagguacuga......................................................................   1      1    Mm1
...................uUaguauuacaucagguacuga......................................................................   1      1    Mm1
...................ugaguauuacaucaggGacuga......................................................................   4      1    Mm1
...................ugaguauuaAaucagguacuga......................................................................   1      1    Mm1
...................ugaguauuacaucaggCacuga......................................................................   6      1    Mm1
...................ugaguauuacaucagguacugaU.....................................................................   189    1    Mm1
...................ugaguauuacaucagguacugaA.....................................................................   33     1    Mm1
....................gaguauuacaucagguacug.......................................................................   4      0    Mm1
.....................aguauuacaucagguacugU......................................................................   1      1    Mm1
......................guauuacaucagguacuga......................................................................   13     0    Mm1
.......................uauuacaucagguacugaU.....................................................................   4      1    Mm1
.....................................................aguaccuucugugauauucau.....................................   2      0    Mm1
.....................................................aguaccuucugugauauucaua....................................   32     0    Mm1
.....................................................aguaccuucuUugauauucaua....................................   1      1    Mm1
```
